# A 15-Minute Exposure to Locally Available Disinfectants Eliminates *Escherichia coli* from Farm-Grown Lettuce While Preserving Quality in Ghana

**DOI:** 10.3390/tropicalmed10100288

**Published:** 2025-10-10

**Authors:** Emmanuel Martin Obeng Bekoe, Gerard Quarcoo, Olga Gonocharova, Divya Nair, Obed Kwabena Offe Amponsah, Karyn Ewurama Quansah, Ebenezer Worlanyo Wallace-Dickson, Emmanuel Tetteh-Doku Mensah, Regina Ama Banu, Mark Osa Akrong, Rony Zachariah

**Affiliations:** 1 Department of Environmental Biology, Biotechnology and Health, Council for Scientific and Industrial Research—Water Research Institute, Accra P.O. Box AH 38, Ghana; gquarcoo@csir.org.gh (G.Q.); reginamabanu@csir.org.gh (R.A.B.); markosaakrong@csir.org.gh (M.O.A.); 2 National Center of Phthisiology, 90 Akhunbaev Str., Bishkek 720000, Kyrgyzstan; gonocharova.ncph@gmail.com; 3 Independent Researcher, Scottsdade, AZ 85259, USA; divsnair08@gmail.com; 4 Department of Pharmacy Practice, Kwame Nkrumah University of Science and Technology, Kumasi P.O. Box LG 25, Ghana; okoamponsah@knust.edu.gh; 5 Environmental Quality and Laboratory Services, Environmental Protection Authority, Accra P.O. Box MB 326, Ghana; ebenezer.worlanyo@epa.gov.gh; 6 Department of Fisheries and Aquaculture, Council for Scientific and Industrial Research—Water Research Institute, Accra P.O. Box AH 38, Ghana; etdmensah@csir.org.gh; 7 UNICEF, UNDP, World Bank, WHO, Special Programme for Research and Training in Tropical Diseases, CH-1211 Geneva, Switzerland; zachariahr@who.int

**Keywords:** SORT IT, operational research, One Health, antibiotic resistant *E. coli*, outbreaks, leafy vegetables

## Abstract

We evaluated the effectiveness of three locally available disinfectants in reducing *Escherichia coli* (*E. coli*) contamination of wastewater-irrigated lettuce while preserving structural integrity. We conducted a quasi-experimental study using lettuce from two farms (Accra and Tamale) in Ghana. Disinfectants tested included (i) salt combined with vinegar, (ii) sequential salt and potassium permanganate, and (iii) sequential vinegar and potassium permanganate. Structural integrity (stem crispness and leaf mushiness) was assessed at 5, 10, and 15 min. *E. coli* counts and antibiotic resistance were determined pre- and post-disinfection. All three disinfectants preserved structural integrity of lettuce at 5 and 10 min. At 15 min, sequential disinfectants preserved 100% structural integrity, while the salt–vinegar mix caused mushiness in 16%. Pre-disinfection *E. coli* counts were 9720 cfu/g for Accra (Inter Quartile range, IQR: 3915–14,175) and 72 cfu/g (IQR: 36–189) for Tamale. All disinfectants eliminated *E. coli* after 15 min. Multi-drug-resistant isolates were common (45% in Accra and 30% in Tamale), particularly against “Watch, restricted use” antibiotics. A 15 min exposure of lettuce to locally available disinfectants, particularly when used sequentially, can eliminate *E. coli* contamination while preserving structural quality. This practical, low-cost intervention can empower households, vendors, and farmers to limit lettuce-borne diarrheal diseases and antimicrobial resistance transmission.

## 1. Introduction

Lettuce (*Lactuca sativa*) is a leafy green vegetable that graces dinner tables around the world. In Ghana, favorable soil and climatic conditions support year-round cultivation, with farmers harvesting up to 10 times annually and earning a 145% return on investment compared to other leafy vegetables [1].

Due to dwindling freshwater resources in Ghana, wastewater, mainly from drains, ponds and streams, is becoming the main irrigation source for lettuce farms [2,3,4]. These sources contain human and animal wastes, leading to contamination with bacteria such as *Escherichia coli* (*E. coli*), *Aeromonas hydrophila*, *Salmonella* species and *Pseudomonas aeruginosa* and subsequent ‘farm-to-fork’ transmission and diarrheal infections [5,6,7,8].

Quarcoo et al. [9], through the Structured Operational Research and Training Initiative (SORT IT), conducted a study in 2022 which showed that lettuce irrigated with wastewater across Ghana was contaminated with multi-drug-resistant *E. coli*. These findings were disseminated to various stakeholders (Table S1), leading to several recommendations (Table S2) including educating farmers and consumers, advocating for farmers to use personal protective wear, adopting drip and furrow irrigation methods, assessing the quality of public water supply systems and formulating food safety standards for leafy vegetables.

However, recognizing the resource-intensity of implementing these recommendations and associated delays, the Council for Scientific and Industrial Research—Water Research Institute (CSIR–WRI) also recommended exploring the use of easy-to-implement disinfection methods for lettuce as a transitional measure.

Salt, vinegar, and potassium permanganate are readily available on the market and can be used to disinfect lettuce and other vegetables. A salt solution creates an osmotic effect, dehydrating microbes while also removing dirt from the lettuce surface. Vinegar, being acidic, lowers the pH and kills bacteria and fungi. Potassium permanganate is a strong oxidizing agent which damages microbial cell walls, leading to their destruction [10,11,12]. Using these disinfectants either in combination or sequentially may have an additive effect, enhancing the overall disinfection of lettuce. If demonstrated to be microbiologically effective, these methods would be valuable for improving lettuce safety at the consumer level.

A PUBMED search revealed that studies conducted in Ghana and elsewhere primarily examined disinfectants used individually, none of them achieved complete (100%) decontamination [10,12,13]. Moreover, no studies reported on the combined or sequential use of salt, vinegar and potassium permanganate to reduce or eliminate bacterial loads. Additionally, there are no studies on the effect of disinfectant exposure duration on the structural integrity of lettuce. Such information is essential to balance effective disinfection with preservation of lettuce quality at the consumer level.

We aimed to evaluate the effectiveness of three locally available solutions applied in combination or sequentially for disinfecting farm-grown lettuce in Ghana. Using samples from wastewater-irrigated farms (Accra and Tamale), our specific objectives were as follows:

(a) We set out to evaluate the structural integrity of the lettuce stem and leaves following exposure to disinfectants across 5, 10, and 15 min, (b) assess *E. coli* counts pre- and post-disinfection with three approaches (salt combined with vinegar, sequential salt and potassium permanganate, and sequential vinegar and potassium permanganate), and (c) determine antibiotic resistance patterns prior to disinfection.

## 2. Materials and Methods

### 2.1. Study Design

A quasi-experimental study was conducted, utilizing laboratory data from lettuce samples.

### 2.2. General Setting

Ghana is situated in West Africa, with Greater Accra as its capital. The country has an estimated population of approximately 34.5 million [14], and the climate is characterized by rainy and dry seasons. Accra is one of the fastest-growing cities in Africa, and Tamale in Northern Ghana is the third-fastest-growing city in the country. Accra hosts about 18% (estimated 5,455,692 persons) of Ghana’s population, whereas Tamale has 374,711 inhabitants [15].

### 2.3. Specific Setting and Study Sites

The study was conducted at vegetable farms in Accra (Greater Accra region) and Tamale (Northern region), where irrigation commonly relies on open drains, streams and ponds.

Watering hoses, cans and sprinklers are the predominant irrigation techniques used at these sites. Lettuce is a commonly cultivated crop in both locations, typically reaching maturity in about 30 days, when the leaves are 4–6 inches tall depending on the variety. Once harvested, lettuce is sold on open markets or directly to traders.

### 2.4. Sample Collection and Storage

Mature lettuce samples were randomly collected from farm sites immediately prior to harvest. The samples were placed in sterile whirl pack bags and transported in a cool box to the laboratory within one hour. A total of 25 composite lettuce samples were prepared for analyses.

### 2.5. Structural Integrity Assessment of Lettuce Before Exposure to Disinfectants

Disinfectant recipes and procedures for lettuce disinfection in Accra and Tamale are shown in Table 1. The three disinfectants were chosen on the basis of two criteria, namely for their low cost and easy availability on the Ghanaian market. Unlike the salt + vinegar combination, the salt + potassium permanganate and vinegar + potassium permanganate treatments must be applied sequentially, as these latter disinfectants react chemically when mixed, reducing their effectiveness.

Three sets of approximately 25 g of lettuce leaves collected from the most mature outer layer of the lettuce plant were rinsed under running tap water and immersed in three sterile bowls, each containing approximately 225 mL of disinfectant solution arranged in a line array. The procedure was conducted separately for both the combined and sequential disinfectant exposures.

Following exposure of lettuce at exposure times of 5, 10, and 15 min, lettuce quality was assessed by (a) manually assessing the crispness of the lettuce by breaking the stem and (b) visually inspecting for mushy leaves. Assessments were conducted together by three investigators—the principal investigator, a senior microbiologist and a lettuce consumer—with consensus used to determine the final quality outcome.

### 2.6. E. coli Identification and Antibiotic Susceptibility Testing

In the laboratory, 25 g of lettuce was weighed and placed into a sterile bag, and 225 mL peptone water was added. The bag was well shaken, and the leaves were massaged to thoroughly dislodge target bacteria. A ten-fold serial dilution of the homogenate was filtered using membrane filtration as previously described in Banu et al. [16]. Post-disinfection *E. coli* counts were processed by aliquoting 1000 µL of the exposed lettuce in an independent rinse constituted with sterile 0.1% tween 80 solution. The Tryptone Bile X-glucuronide medium (TBX) (Oxoid, Hampshire, UK) was adapted for *E. coli* detection and colony counts.

Three presumptive isolates were randomly selected from each plate and sub-cultured on nutrient agar for antibiotic susceptibility testing using the Kirby–Bauer disk diffusion method, as described by the Clinical Laboratory Standards Institute (CLSI) guidelines [17]. The zones of inhibition were measured in millimeters and recorded for the selected antibiotics (gentamicin 10 µg (aminoglycosides); cefuroxime 30 µg (second-generation cephalosporins); trimethoprim–sulfamethoxazole 1.25/23.75 µg (sulfonamide–trimethoprim combinations); aztreonam 15 µg (monobactam); ceftriaxone 30 µg (third-generation cephalosporins); ertapenem 10 µg (carbapenem); and chloramphenicol 30 µg (amphenicols).

### 2.7. Quality Control Procedures

Sterile distilled water was used as a negative control during bacterial analysis. The reference organism *E. coli* ATCC 25922 was utilized as a positive control according to CLSI guidelines. All bacterial analyses were carried out in the laminar workstation under aseptic conditions.

### 2.8. Study Inclusion and Period

Mature lettuce samples were randomly collected from farm sites and analyzed in April 2025.

### 2.9. Data Collection, Variables, and Validation

Information on sample collection sites, leaf structural integrity, type of disinfectant solution, exposure time to disinfectants (5, 10, and 15 min), *E. coli* load, and resistance profiles was recorded in a laboratory register and subsequently transferred to Microsoft Excel spreadsheet maintained in the laboratory. Data entry was performed by the principal investigator and a trained data assistant. To ensure accuracy, all entries in the Microsoft excel file were cross-checked against the raw data in the laboratory register.

### 2.10. Statistical Analyses

Descriptive statistics (counts and frequencies) including measures of central tendency (median, inter-quartile ranges (IQRs)) are used to report the results in tables. Analysis was performed using Microsoft Excel (2019 version) and Open Epi (version 3.01). The level of significance was set at *p* ≤ 0.05, with 95% confidence intervals used throughout.

## 3. Results

### 3.1. Structural Integrity of Lettuce

Table 2 presents the structural integrity of the lettuce stem and leaves following exposure to disinfectants across times of 5, 10, and 15 min. At both 5 and 10 min, all three disinfectant solutions preserved lettuce stem crispness. After 15 min of sequential application of disinfectants, there was full structural preservation of the lettuce stem and leaves. In contrast, the combined use of salt + vinegar resulted in mushiness in 16% (4/25) of samples.

### 3.2. E. coli Counts Pre- and Post-Disinfection with Three Different Solutions

In Accra, pre-disinfection *E. coli* counts were markedly higher (median: 9720 cfu/g; IQR: 3915–14,175) compared with Tamale (median: 72 cfu/g, IQR: 36–189). After 15 min of exposure to any of the three disinfectants, *E. coli* was eliminated (Table 3).

### 3.3. Antibiotic Resistance of E. coli Exposure of Lettuce to Disinfectants

Table 4 presents the antibiotic resistance patterns of *E. coli* isolates prior to lettuce disinfection. Multi-drug resistance was common at both sites, with higher resistance in Accra, notably against WHO “Watch or restricted use” category antibiotics. Overall, multi-drug resistance was 45% in Accra and 30% in Tamale. No resistance was detected in reserve-category antibiotics (Aztreonam).

## 4. Discussion

This is the first study from Ghana to simultaneously assess the microbial effectiveness of locally available, low-cost, household-level disinfectants and their impact on lettuce quality. The findings demonstrate that all three disinfection methods—combined salt and vinegar solution, sequential salt and potassium permanganate, and sequential vinegar and potassium permanganate—eliminated *E. coli* contamination after 15 min of exposure. Sequential application of disinfectants preserved the full structural integrity of lettuce stems and leaves across all exposure times (5, 10, and 15 min). In contrast, the combined salt–vinegar solution, while maintaining stem structure, resulted in a mushy leaf texture in 16% of samples at 15 min. These results suggest that sequential disinfection methods are preferable for balancing leaf quality with antimicrobial effectiveness. Pre-disinfection analysis revealed high *E. coli* loads in lettuce and multi-drug resistance—a pattern consistent with other studies from Ghana, United Arab Emirates and Nigeria [9,18,19]

The findings of this study have significant implications for food safety in Ghana and similar settings where dwindling freshwater resources often force farmers to rely on wastewater for agricultural irrigation [20,21]. The detection of multi-resistant *E. coli* on lettuce prior to disinfection highlights its potential role as a vehicle for diarrheal disease transmission and for the spread of antimicrobial resistance to farmers, consumers, and the wider community. This study demonstrates that locally available, inexpensive disinfectant solutions offer a simple and practical approach to improving lettuce safety before consumption.

The strengths of the study are as follows: (a) it was conducted by the Council for Scientific and Industrial Research, which has experienced researchers and quality-controlled laboratories; (b) the findings reflect real-world operational realities, as lettuce samples were harvested from farm sites; (c) both disinfectant efficacy and preservation of lettuce quality were assessed, enhancing the practical applicability and acceptability of the findings; (d) structural integrity was evaluated post-disinfection by consensus, including input from a consumer; and (e) reporting adhered to the Strengthening The Reporting of Observational Studies in Epidemiology (STROBE) guidelines [22]. Importantly, this study addressed a nationally identified One Health research priority in antimicrobial resistance (AMR), supporting its potential for policy relevance and research uptake.

The study has a few limitations. Firstly, it was conducted on only two farm sites and limited to a single month (April), which means it may not capture seasonal variations or represent *E. coli* loads from other regions. Further expansion of study sites should be considered. Furthermore, disinfectant effectiveness was assessed solely against *E. coli* and not against other WHO priority organisms, such as *Shigella* and *Salmonella*, or helminths, which may also be present in wastewater. These aspects merit further specific research.

The study findings have a number of policy and practice implications. First, lettuce samples were contaminated with *E. coli*, with substantially higher loads observed in Accra compared to Tamale. This difference may be attributed to Accra’s higher population density [23] and consequently, the greater contamination from the wastewater used for irrigation. Any of the three disinfection methods evaluated in this study could be applied for disinfecting lettuce. However, a prior step would be to develop clear, user-friendly guidelines on disinfectant use, including safe concentrations, appropriate exposure times, and instructions in local languages. These guidelines could be disseminated via public health campaigns and community and farmer associations. An operational consideration is the optimal point for making disinfectants available. A practical approach would be to provide both disinfectants and user guidelines at lettuce sales points, including farm sites (at times of harvest) and at marketplaces. Such a strategy could bring additional economic opportunities for farmers and small-scale market vendors.

Secondly, at 15 min, all three disinfectants eliminated *E. coli* contamination. However, the combined salt–vinegar solution, while maintaining stem crispness, caused some loss of leaf integrity at 15 min. This notwithstanding, in contexts where there is scarcity of clean water to rinse off potassium permanganate from lettuce leaves, this would be the preferred option, as both salt and vinegar are common constituents of salads. Although the slight loss of leaf integrity may remain acceptable to consumers, a qualitative study would be valuable to explore consumer acceptability of lettuce treated with different disinfectant solutions.

Additionally, further assessment of *E. coli* elimination at 10 min with the three disinfectant solutions would be worthwhile. If complete decontamination can be achieved with this shorter exposure time, it could be set as the household disinfection standard for lettuce disinfection, balancing microbial safety with the preservation of structural quality.

Finally, global antimicrobial resistance surveillance systems—such as the WHO’s Global Antimicrobial Resistance Surveillance System (GLASS)—have primarily focused on human health [24]. The high prevalence of antibiotic-resistant *E. coli*, including the multi-drug-resistant strains identified in this study, underscores the urgent need to expand and integrate AMR surveillance to include foodborne and environmental bacteria. *E. coli* resistance to potassium permanganate is anticipated, and this too could be considered for surveillance [25]. Adopting such a broader One Health approach would provide a more accurate understanding of AMR dynamics across human, animal, and environmental sectors and inform targeted interventions in One Health [26,27].

## 5. Conclusions

In conclusion, this study demonstrates that a 15 min exposure of farm-grown lettuce to locally available disinfectants, particularly when used sequentially (sequential salt and potassium permanganate and sequential vinegar and potassium permanganate), can effectively eliminate *E. coli* contamination while preserving structural quality. It offers a feasible solution that can empower households, vendors, and farmers to limit lettuce-borne diarrheal disease and AMR transmission.

Furthermore, the use of such disinfectants can serve as a stopgap (safety-net) measure for protecting public health while Ghana makes broader efforts towards tackling its water and sanitation challenges.

## Figures and Tables

**Table 1:** Disinfectant types, composition and disinfection procedures for lettuce in Accra and Tamale vegetable farms, Ghana (April 2025).

Disinfectants	Composition	Disinfection Procedure
1. Salt and vinegar mixed together	3% salt solution mixed with white 5% vinegar (1 part to 3 parts water)	Rinse the lettuce with sterile water. Soak the lettuce in the recipe for 5, 10 or 15 min. Rinse thoroughly with sterile water.
2. Salt and potassium permanganate used sequentially	3% salt solution 0.01% potassium permanganate	Rinse the lettuce with sterile water. Soak the lettuce in the salt solution for the exposure times ^1^; then, rinse thoroughly with sterile water. Then, soak in the potassium permanganate solution for the exposure times ^1^, and finally, rinse with sterile water.
3. Vinegar and potassium permanganate used sequentially	White 5% vinegar (1 part to 3 parts water) 0.01% Potassium Permanganate	Rinse the lettuce with sterile water. Soak the lettuce in the vinegar solution for the exposure time ^1^; then, rinse thoroughly with sterile water. Then, soak in the potassium permanganate solution for the exposure time ^1^, and finally, rinse with sterile water.

^1^ Exposure time 5 min: immersion of lettuce leaves in the first solution for 2.5 min, followed by the second solution for 2.5 min. Exposure time 10 min: immersion of lettuce leaves in the first solution for 5 min, followed by the second solution for 5 min. Exposure time 15 min: immersion of lettuce leaves in the first solution for 7.5 min, followed by the second solution for 7.5 min.

**Table 2:** Structural integrity of the lettuce stem and leaves following exposure to disinfectants for 5, 10, and 15 min (Accra and Tamale, Ghana) (April 2025).

Disinfectant Solutions	Exposure Duration											
	5 Min				10 Min				15 Min			
	Lettuce Stem Crispness Maintained		Mushy Leaves		Lettuce Stem Crispness Maintained		Mushy Leaves		Lettuce Stem Crispness Maintained		Mushy Leaves	
	*n*	(%)	*n*	(%)	*n*	(%)	*n*	(%)	*n*	(%)	*n*	(%)
Salt and vinegar mix	25	100	0	0	25	100	0	0	25	100	4	16
Sequential use of salt and potassium permanganate	25	100	0	0	25	100	0	0	25	100	0	0
Sequential use of vinegar and potassium permanganate	25	100	0	0	25	100	0	0	25	100	0	0

**Table 3:** *E. coli* counts pre- and post-disinfection of lettuce using three different solutions for 15 min (Accra and Tamale, Ghana) (April 2025).

Sampled Areas	Disinfecting Solutions	Pre-Disinfection *E. coli* Counts (cfu/g) Median (IQR)	Post-Disinfection *E. coli* Counts (cfu/g) Median (IQR)
Accra	1. Salt and vinegar mixed	9720 (3915–14,175)	0
	2. Sequential use of salt and potassium permanganate		0
	3. Sequential use of vinegar and potassium permanganate		0
Tamale	1. Salt and vinegar mixed	72 (36–189)	0
	2. Sequential use of salt and potassium permanganate		0
	3. Sequential use of vinegar and potassium permanganate		0

**Table 4:** Antibiotic resistance of *E. coli* in lettuce before disinfection, Accra and Tamale, Ghana (April 2025).

AWaRe Categories *	Accra N = 42	Tamale N = 27
	*n* (%, 95% CI)	*n* (%, 95% CI)
*Access antibiotics*		
Gentamicin	6 (14, 6–27)	0
Chloramphenicol 30 µg	6 (14, 6–27)	0
Trimethoprim—sulfamethoxazole 1.25/23.75 µg	12 (28, 16–44)	1 (3, 0.2–17)
*Watch antibiotics*		
Ceftriaxone 30 µg	21 (50, 35–65)	3 (18, 3–27)
Cefuroxime 30 µg	30 (71, 56–84)	26 (96, 83–100)
Ertapenem 10 µg	26 (61, 47–76)	3 (11, 3–27)
*Reserve antibiotics*		
Aztreonam 15 µg	0	0
*Multi-drug resistance* (≥3 antibiotic classes)	19 (45, 31–60)	8 (30, 15–49)

* The AWaRe classification is a system developed by the World Health Organization to promote antibiotic stewardship. **Access**—first-line antibiotics, widely available; **Watch**—higher resistance risk, restricted use; **Reserve**—last-resort options for multi-drug-resistant infections.

## Data Availability

Requests to access these data should be sent to the corresponding author.

## Supplementary Materials

The following supporting information can be downloaded at https://www.mdpi.com/article/10.3390/tropicalmed10100288/s1. Table S1: Summary of dissemination strategies and actions taken following Quarcoo et al., 2022 study [9]; Table S2: List of recommendations, action status and details of action on assessing bacterial contamination of lettuce conducted by Quarcoo et al. [9].
