# Supplementary material for: A 15-Minute Exposure to Locally Available Disinfectants Eliminates Escherichia coli from Farm-Grown Lettuce While Preserving Quality in Ghana
Source: Trop Med Infect Dis. 2025 Oct 10;10(10):288. doi: 10.3390/tropicalmed10100288 (PMC12568300; doi:10.3390/tropicalmed10100288)
Supplement: Supplementary file 1 [file tropicalmed-10-00288-s001.zip › Supplementary 1_Revised.pdf]

Supplementary 1

Table S1:Summary of dissemination strategies and actions taken following Quarcoo et al., 2022 study

Abbreviations: AMR – Antimicrobial resistance; IJEPH - International Journal of Environmental Research and Public Health; KNUST - Kwame Nkrumah University of Science and Technology; SORT IT – Structured

How	To whom	Where (numbers)	When
Planning for dissemination (to whom, where, when?)	Study team and WHO-Ghana office<br>CSIR - WRI team	SORT IT workshop, Aqua Safari Eco Resort, Ada (20)<br>CSIR - WRI Conference facility (15)	October, 2022
Publication in a scientific journal	IJEPH readership<br>Department of Theoretical and Applied Biology of KNUST<br>AMR National committee	[Quarcoo et al., (2022)]<br>(2193 article views; 17 citations; 244 reads)<br>WhatsApp group (80)	October, 2022
PowerPoint presentations	Research and Technical staff of CSIR - WRI, Tamale	WRI Conference facility (20)	April, 2023
Plain language hand-outs	EBBH Divisional Team<br>Ministry of Food and Agriculture (MoFA) and farmers<br><br>SORT IT workshop members and WHO - Ghana office,<br><br>Consumers	CSIR-WRI Accra and Tamale (20)<br>Ayawaso West Municipal, Accra and Tamale Metropolis Directorate (32)<br><br>Oak Plaza Hotel, Accra (36)<br><br>Church youth groups in Accra and Tamale (48)	November, 2022<br>March to July 2023<br><br>August - September, 2023
Publication uploaded on websites	National and international stakeholders/general public	CSIR website - [CSIR AND WHO]<br>WHO country website - [3073-tdr-update-tdr-sort-it-map-161221.jpg (1501×786) (who.int)]<br>TDR websites - [SORT IT operational research and training (who.int)]	July, 2023
Conferences	Researchers and students	Noguchi Annual Research meeting, Accra (> 400)<br><br>3rd International Scientific Congress by the African Association for Research and Control of Antimicrobial Resistance, Cote D'Ivoire (>100)	November, 2022<br><br>February, 2023

Operational Research and Training Initiative; WHO - World Health Organization; Environmental Biology, Biotechnology and Health; TDR - Special Programme for Research and Training in Tropical Diseases
