# Supplementary material for: A 15-Minute Exposure to Locally Available Disinfectants Eliminates Escherichia coli from Farm-Grown Lettuce While Preserving Quality in Ghana
Source: Trop Med Infect Dis. 2025 Oct 10;10(10):288. doi: 10.3390/tropicalmed10100288 (PMC12568300; doi:10.3390/tropicalmed10100288)
Supplement: Supplementary file 1 [file tropicalmed-10-00288-s001.zip › Supplementary 2.pdf]

Supplementary 2

Table S2: List of recommendations, action status and details of action on assessing bacterial contamination of lettuce conducted by Quarcoo et al., (2022) [1]

Recommendations	Action status	Details of action (when & what)
Inform consumers on bacterial contamination of lettuce	Implemented	*October/2022* to *December/2023*<br>IEC to consumers in both Accra and Tamale<br>The published manuscript was circulated on social media, research platforms i.e: (ResearchGate and Google scholar)<br>Oral presentations were made at local and International conferences
Advocate for personal protective wear (e.g., gloves, gumboots) for farmers	Partially implemented due to lack of funds	Some farmers who could afford protective wear have begun its use.
Assess quality of public water supply systems	Implemented	*Oct. 2022 - March 2023*<br>Public Utility Regulatory Commission (PURC) has undertaken a nation-wide surveillance of public water supply.
The adaption of drip or furrow methods of irrigation	Partially implemented	*March/2023*<br>Some richer farmers partially use these irrigation methods.<br>No funding for the large-scale adaption of this recommendation.
Explore point-of-consumption disinfection methods for decontamination (proposed after publication of Quarcoo et al., 2022 study	New research study proposed	*July 2024*<br>New study endorsed and launched by CSIR, WHO Country office and TDR.
Formulate standards for fresh leafy vegetable food safety	Under consideration	*On-going*<br>Standards for regulating ready-to- eat food exist, but standards for fresh produce are under development.

REFERENCE

1. Quarcoo, G.; Adomako, L.A.B.; Abrahamyan, A.; Armoo, S.; Sylverken, A.A.; Addo, M.G.; Alaverdyan, S.; Jessani, N.S.; Harries, A.D.; Ahmed, H.; et al. What Is in the Salad? *Escherichia coli* and Antibiotic Resistance in Lettuce Irrigated with Various Water Sources in Ghana. *Int J Environ Res Public Health* **2022**, *19*, 1–12, doi:https:// doi.org/10.3390/ijerph191912722.
